# Morpholin-4-ium 4-meth­oxy­benzoate 4-meth­oxy­benzoic acid monohydrate

**DOI:** 10.1107/S1600536811023361

**Published:** 2011-06-22

**Authors:** Li-Ping Feng, Liang Zhao

**Affiliations:** a Department of Chemical & Environmental Engineering, Anyang Institute of Technology, Anyang 455000, People’s Republic of China

## Abstract

In the crystal structure of the title compound, C_4_H_10_NO^+^·C_8_H_7_O_3_^−^·C_8_H_8_O_3_·H_2_O, cations, anions and neutral mol­ecules are linked by inter­molecular N—H⋯O and O—H⋯O hydrogen bonds into chains running parallel to the *c* axis. The  –CO_2_ groups make dihedral angles of 4.6 (3) and 5.7 (4)° with the attached ring in the 4-methoxybenzoic acid molecule and the 4-methoxybenzoate anion, respectively.

## Related literature

For related studies on co-crystals of amino derivatives, see: Fu *et al.* (2010 ▶); Aminabhavi *et al.* (1986 ▶).
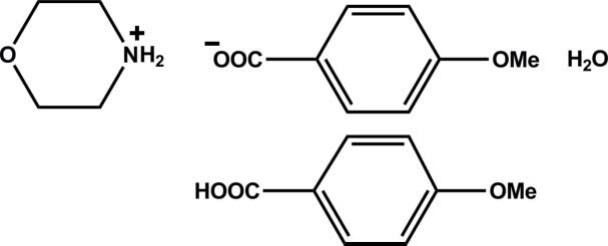


## Experimental

### 

#### Crystal data

C_4_H_10_NO^+^·C_8_H_7_O_3_^−^·C_8_H_8_O_3_·H_2_O
*M* *_r_* = 409.43
Monoclinic, 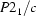

*a* = 21.874 (4) Å
*b* = 11.753 (2) Å
*c* = 8.3618 (17) Å
β = 100.63 (3)°
*V* = 2112.9 (7) Å^3^
*Z* = 4
Mo *K*α radiation
μ = 0.10 mm^−1^
*T* = 298 K
0.30 × 0.05 × 0.05 mm

#### Data collection

Rigaku Mercury2 diffractometer
Absorption correction: multi-scan (*CrystalClear*; Rigaku, 2005 ▶) *T*_min_ = 0.910, *T*_max_ = 1.000
21489 measured reflections
4842 independent reflections
2453 reflections with *I* > 2σ(*I*)
*R*_int_ = 0.077

#### Refinement

*R*[*F* ^2^ > 2σ(*F* ^2^)] = 0.063
*wR*(*F* ^2^) = 0.162
*S* = 1.03
4842 reflections
264 parameters
H-atom parameters constrained
Δρ_max_ = 0.16 e Å^−3^
Δρ_min_ = −0.23 e Å^−3^

### 

Data collection: *CrystalClear* (Rigaku, 2005 ▶); cell refinement: *CrystalClear*; data reduction: *CrystalClear*; program(s) used to solve structure: *SHELXS97* (Sheldrick, 2008 ▶); program(s) used to refine structure: *SHELXL97* (Sheldrick, 2008 ▶); molecular graphics: *SHELXTL* (Sheldrick, 2008 ▶); software used to prepare material for publication: *SHELXTL*.

## Supplementary Material

Crystal structure: contains datablock(s) I, global. DOI: 10.1107/S1600536811023361/rz2614sup1.cif

Structure factors: contains datablock(s) I. DOI: 10.1107/S1600536811023361/rz2614Isup2.hkl

Supplementary material file. DOI: 10.1107/S1600536811023361/rz2614Isup3.cml

Additional supplementary materials:  crystallographic information; 3D view; checkCIF report

## Figures and Tables

**Table 1:** Hydrogen-bond geometry (Å, °)

*D*—H⋯*A*	*D*—H	H⋯*A*	*D*⋯*A*	*D*—H⋯*A*
N1—H1*A*⋯O5^i^	0.90	1.75	2.628 (3)	164
N1—H1*B*⋯O2	0.90	1.96	2.837 (3)	163
O1—H1⋯O1*W*^i^	0.82	1.76	2.579 (2)	173
O1*W*—H1*WA*⋯O4	0.82	1.91	2.714 (2)	167
O1*W*—H1*WB*⋯O4^ii^	0.82	1.91	2.712 (3)	164

Symmetry codes: (i) 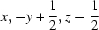; (ii) 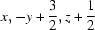.

**Fig. 1.**
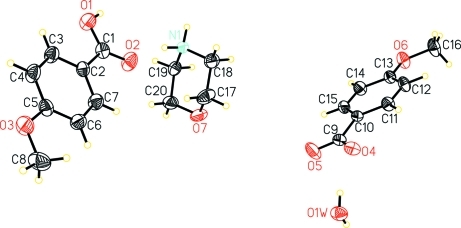
The molecular structure of the title compound with displacement ellipsoids drawn at the 30% probability level.

**Fig. 2.**
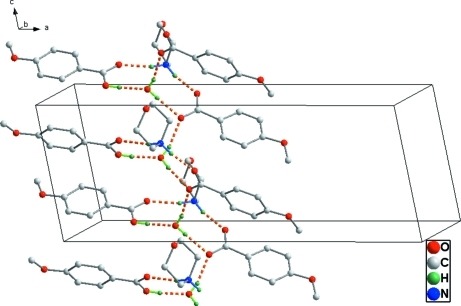
The crystal packing of the title compound showing a one-dimensional chain (dashed line). Hydrogen atoms not involved in hydrogen bonding (dashed lines) are omitted for clarity.

## supplementary crystallographic information

### Comment

The amino derivatives have found wide range of applications in material science, such as magnetic, fluorescent and dielectric behaviors, and there has been an increasing interest in the preparation of amino co-crystal compounds (Aminabhavi *et al.,* 1986; Fu, *et al.* 2010). We report here the crystal structure of the title compound, morpholin-4-ium 4-methoxybenzoate 4-methoxybenzoic acid monohydrate.

The asymmetric unit of the title commpound is composed of one 4-methoxybenzoate anion, one morpholin-4-ium cation, one 4-methoxybenzoic acid molecule and one water molecule (Fig. 1). The morpholine ring is in a chair conformation. All geometric parameters are in the normal ranges. In the crystal structure, the H atoms bound to N and O atoms are involved in intermolecular N—H···O and O—H···O hydrogen bonds (Table 1) linking ions and neutral molecules into one-dimensional chains parallel to the *c*-axis (Fig. 2).

### Experimental

A mixture of morpholine (0.4 mmol) 4-methoxybenzoic acid (0.8 mmol) was dissolved in distilled water (10 ml). Colourless crystals suitable for X-ray analysis were obtained after 3 days on slow evaporation of the solvent.

### Refinement

All H atoms attached to C atoms were fixed geometrically and treated as riding, with C–H = 0.93-0.97 Å and with *U*_iso_(H) = 1.2*U*_eq_(C) or 1.5*U*_eq_(C) for methyl H atoms. The amine and carboxylic H atoms were located in a difference Fourier map and refined as riding, with the N–H = 0.90 (2) Å, O–H = 0.82 (2) Å, and with *U_iso_*(H) = 1.2*U_eq_*(N) or 1.5*U_eq_*(O).

### Crystal data

**Table:**

C_4_H_10_NO^+^·C_8_H_7_O_3_^−^·C_8_H_8_O_3_·H_2_O	*F*(000) = 872
*M**_r_* = 409.43	*D*_x_ = 1.287 Mg m^−^^3^
Monoclinic, *P*2_1_/*c*	Mo *K*α radiation, λ = 0.71073 Å
Hall symbol: -P 2ybc	Cell parameters from 4842 reflections
*a* = 21.874 (4) Å	θ = 3.0–27.5°
*b* = 11.753 (2) Å	µ = 0.10 mm^−^^1^
*c* = 8.3618 (17) Å	*T* = 298 K
β = 100.63 (3)°	Needle, colourless
*V* = 2112.9 (7) Å^3^	0.30 × 0.05 × 0.05 mm
*Z* = 4	

### Data collection

**Table:**

Rigaku Mercury2 diffractometer	4842 independent reflections
Radiation source: fine-focus sealed tube	2453 reflections with *I* > 2σ(*I*)
graphite	*R*_int_ = 0.077
Detector resolution: 13.6612 pixels mm^-1^	θ_max_ = 27.5°, θ_min_ = 3.0°
CCD profile fitting scans	*h* = −28→27
Absorption correction: multi-scan (*CrystalClear*; Rigaku, 2005)	*k* = −15→15
*T*_min_ = 0.910, *T*_max_ = 1.000	*l* = −10→10
21489 measured reflections	

### Refinement

**Table:**

Refinement on *F*^2^	Primary atom site location: structure-invariant direct methods
Least-squares matrix: full	Secondary atom site location: difference Fourier map
*R*[*F*^2^ > 2σ(*F*^2^)] = 0.063	Hydrogen site location: inferred from neighbouring sites
*wR*(*F*^2^) = 0.162	H-atom parameters constrained
*S* = 1.03	*w* = 1/[σ^2^(*F*_o_^2^) + (0.058*P*)^2^ + 0.3115*P*] where *P* = (*F*_o_^2^ + 2*F*_c_^2^)/3
4842 reflections	(Δ/σ)_max_ < 0.001
264 parameters	Δρ_max_ = 0.16 e Å^−^^3^
0 restraints	Δρ_min_ = −0.23 e Å^−^^3^

### Special details

**Table:**

Geometry. All esds (except the esd in the dihedral angle between two l.s. planes) are estimated using the full covariance matrix. The cell esds are taken into account individually in the estimation of esds in distances, angles and torsion angles; correlations between esds in cell parameters are only used when they are defined by crystal symmetry. An approximate (isotropic) treatment of cell esds is used for estimating esds involving l.s. planes.
Refinement. Refinement of F^2^ against ALL reflections. The weighted R-factor wR and goodness of fit S are based on F^2^, conventional R-factors R are based on F, with F set to zero for negative F^2^. The threshold expression of F^2^ > 2sigma(F^2^) is used only for calculating R-factors(gt) etc. and is not relevant to the choice of reflections for refinement. R-factors based on F^2^ are statistically about twice as large as those based on F, and R- factors based on ALL data will be even larger.

### Fractional atomic coordinates and isotropic or equivalent isotropic displacement parameters (Å^2^)

**Table:**

	*x*	*y*	*z*	*U*_iso_*/*U*_eq_	
O4	0.30003 (8)	0.67567 (15)	0.28036 (19)	0.0575 (5)	
C10	0.40047 (10)	0.61009 (19)	0.2544 (3)	0.0400 (5)	
O6	0.56764 (7)	0.62620 (15)	0.0900 (2)	0.0653 (5)	
C12	0.46359 (11)	0.6996 (2)	0.0826 (3)	0.0517 (6)	
H12A	0.4682	0.7552	0.0067	0.062*	
C14	0.50318 (12)	0.5405 (2)	0.2459 (3)	0.0556 (7)	
H14A	0.5350	0.4887	0.2807	0.067*	
C13	0.51084 (11)	0.6245 (2)	0.1353 (3)	0.0452 (6)	
O5	0.34193 (10)	0.53418 (18)	0.4368 (3)	0.0845 (7)	
C15	0.44880 (12)	0.5339 (2)	0.3038 (3)	0.0497 (6)	
H15A	0.4441	0.4771	0.3780	0.060*	
C11	0.40899 (11)	0.6921 (2)	0.1430 (3)	0.0486 (6)	
H11A	0.3772	0.7438	0.1075	0.058*	
C9	0.34312 (12)	0.6061 (2)	0.3279 (3)	0.0493 (6)	
C16	0.57873 (13)	0.7102 (3)	−0.0241 (4)	0.0756 (9)	
H16A	0.6193	0.6990	−0.0499	0.113*	
H16B	0.5765	0.7846	0.0221	0.113*	
H16C	0.5479	0.7038	−0.1215	0.113*	
O1W	0.23075 (8)	0.74295 (15)	0.5012 (2)	0.0634 (5)	
H1WA	0.2490	0.7132	0.4346	0.095*	
H1WB	0.2567	0.7703	0.5746	0.095*	
N1	0.26721 (9)	0.00361 (15)	0.1437 (2)	0.0462 (5)	
H1A	0.2883	−0.0209	0.0674	0.055*	
H1B	0.2299	−0.0308	0.1336	0.055*	
O1	0.11518 (8)	−0.23024 (16)	0.0259 (2)	0.0703 (6)	
H1	0.1514	−0.2296	0.0125	0.105*	
O7	0.26402 (9)	0.14332 (16)	0.4181 (2)	0.0696 (6)	
C1	0.10461 (12)	−0.1389 (2)	0.1073 (3)	0.0526 (6)	
C2	0.04022 (11)	−0.1286 (2)	0.1359 (3)	0.0469 (6)	
O2	0.14422 (8)	−0.06853 (17)	0.1541 (3)	0.0785 (6)	
C5	−0.08025 (12)	−0.0989 (2)	0.1886 (3)	0.0550 (7)	
O3	−0.14078 (8)	−0.09074 (17)	0.2062 (3)	0.0777 (6)	
C7	0.02520 (12)	−0.0385 (2)	0.2269 (3)	0.0587 (7)	
H7A	0.0560	0.0130	0.2708	0.070*	
C4	−0.06580 (12)	−0.1890 (2)	0.0972 (4)	0.0661 (8)	
H4A	−0.0968	−0.2402	0.0528	0.079*	
C6	−0.03452 (12)	−0.0231 (2)	0.2543 (3)	0.0594 (7)	
H6A	−0.0438	0.0378	0.3167	0.071*	
C3	−0.00622 (11)	−0.2043 (2)	0.0707 (3)	0.0571 (7)	
H3A	0.0029	−0.2657	0.0089	0.069*	
C19	0.30232 (12)	−0.0252 (2)	0.3071 (3)	0.0533 (7)	
H19A	0.3443	0.0047	0.3197	0.064*	
H19B	0.3050	−0.1072	0.3196	0.064*	
C18	0.25775 (15)	0.1276 (2)	0.1291 (3)	0.0706 (8)	
H18A	0.2314	0.1452	0.0255	0.085*	
H18B	0.2975	0.1652	0.1328	0.085*	
C20	0.27063 (14)	0.0242 (2)	0.4338 (3)	0.0671 (8)	
H20A	0.2298	−0.0101	0.4253	0.081*	
H20B	0.2944	0.0063	0.5405	0.081*	
C8	−0.15903 (14)	0.0060 (3)	0.2886 (4)	0.0879 (10)	
H8A	−0.2034	0.0060	0.2798	0.132*	
H8B	−0.1463	0.0740	0.2404	0.132*	
H8C	−0.1396	0.0030	0.4013	0.132*	
C17	0.22811 (15)	0.1700 (2)	0.2644 (4)	0.0769 (9)	
H17A	0.2230	0.2518	0.2550	0.092*	
H17B	0.1872	0.1362	0.2555	0.092*	

### Atomic displacement parameters (Å^2^)

**Table:**

	*U*^11^	*U*^22^	*U*^33^	*U*^12^	*U*^13^	*U*^23^
O4	0.0538 (11)	0.0730 (12)	0.0469 (10)	0.0087 (10)	0.0126 (8)	0.0003 (9)
C10	0.0456 (14)	0.0418 (13)	0.0326 (12)	−0.0019 (11)	0.0079 (11)	−0.0025 (10)
O6	0.0477 (11)	0.0712 (13)	0.0804 (13)	0.0108 (9)	0.0210 (10)	0.0273 (10)
C12	0.0467 (15)	0.0578 (16)	0.0495 (14)	0.0032 (12)	0.0063 (12)	0.0211 (12)
C14	0.0563 (16)	0.0518 (16)	0.0604 (16)	0.0163 (13)	0.0155 (14)	0.0141 (13)
C13	0.0406 (14)	0.0485 (15)	0.0465 (14)	0.0004 (11)	0.0082 (11)	0.0052 (12)
O5	0.0960 (16)	0.0856 (15)	0.0870 (15)	0.0203 (12)	0.0561 (13)	0.0340 (12)
C15	0.0636 (17)	0.0413 (14)	0.0459 (14)	0.0051 (12)	0.0146 (13)	0.0096 (11)
C11	0.0451 (15)	0.0513 (15)	0.0478 (14)	0.0083 (11)	0.0042 (12)	0.0115 (12)
C9	0.0592 (17)	0.0516 (16)	0.0387 (14)	−0.0028 (13)	0.0132 (13)	−0.0047 (12)
C16	0.0621 (19)	0.076 (2)	0.095 (2)	0.0011 (15)	0.0328 (17)	0.0317 (18)
O1W	0.0488 (10)	0.0806 (14)	0.0636 (12)	−0.0085 (9)	0.0179 (9)	−0.0138 (10)
N1	0.0494 (12)	0.0429 (11)	0.0500 (12)	−0.0063 (9)	0.0189 (10)	−0.0085 (9)
O1	0.0494 (11)	0.0678 (13)	0.0960 (15)	−0.0007 (9)	0.0197 (10)	−0.0159 (11)
O7	0.0921 (15)	0.0609 (13)	0.0555 (12)	0.0106 (10)	0.0132 (10)	−0.0171 (10)
C1	0.0443 (15)	0.0538 (17)	0.0588 (16)	0.0016 (13)	0.0075 (13)	0.0077 (13)
C2	0.0421 (14)	0.0471 (15)	0.0507 (14)	−0.0015 (11)	0.0061 (12)	0.0062 (12)
O2	0.0450 (11)	0.0735 (14)	0.1168 (17)	−0.0146 (10)	0.0145 (11)	−0.0202 (12)
C5	0.0444 (15)	0.0568 (17)	0.0662 (17)	−0.0019 (13)	0.0165 (13)	0.0060 (14)
O3	0.0516 (12)	0.0812 (14)	0.1071 (16)	−0.0047 (10)	0.0327 (11)	−0.0130 (12)
C7	0.0471 (16)	0.0622 (17)	0.0655 (17)	−0.0077 (13)	0.0070 (14)	−0.0077 (14)
C4	0.0475 (16)	0.0589 (18)	0.092 (2)	−0.0117 (13)	0.0143 (15)	−0.0122 (16)
C6	0.0594 (18)	0.0560 (17)	0.0636 (17)	−0.0009 (13)	0.0137 (14)	−0.0081 (13)
C3	0.0474 (16)	0.0486 (15)	0.0761 (19)	−0.0017 (12)	0.0137 (14)	−0.0067 (13)
C19	0.0523 (15)	0.0430 (14)	0.0625 (17)	0.0021 (12)	0.0051 (13)	−0.0009 (12)
C18	0.110 (2)	0.0468 (17)	0.0551 (17)	0.0088 (16)	0.0145 (17)	0.0071 (13)
C20	0.091 (2)	0.065 (2)	0.0461 (15)	0.0022 (16)	0.0145 (15)	0.0004 (14)
C8	0.069 (2)	0.108 (3)	0.095 (2)	0.0141 (19)	0.0372 (19)	−0.009 (2)
C17	0.097 (2)	0.0520 (17)	0.079 (2)	0.0267 (16)	0.0081 (18)	−0.0102 (15)

### Geometric parameters (Å, °)

**Table:**

O4—C9	1.256 (3)	C1—O2	1.209 (3)
C10—C11	1.377 (3)	C1—C2	1.476 (3)
C10—C15	1.389 (3)	C2—C7	1.379 (3)
C10—C9	1.496 (3)	C2—C3	1.384 (3)
O6—C13	1.364 (3)	C5—O3	1.362 (3)
O6—C16	1.425 (3)	C5—C6	1.376 (3)
C12—C13	1.369 (3)	C5—C4	1.377 (4)
C12—C11	1.383 (3)	O3—C8	1.424 (3)
C12—H12A	0.9300	C7—C6	1.379 (3)
C14—C15	1.367 (3)	C7—H7A	0.9300
C14—C13	1.384 (3)	C4—C3	1.374 (3)
C14—H14A	0.9300	C4—H4A	0.9300
O5—C9	1.246 (3)	C6—H6A	0.9300
C15—H15A	0.9300	C3—H3A	0.9300
C11—H11A	0.9300	C19—C20	1.487 (3)
C16—H16A	0.9600	C19—H19A	0.9700
C16—H16B	0.9600	C19—H19B	0.9700
C16—H16C	0.9600	C18—C17	1.490 (4)
O1W—H1WA	0.8204	C18—H18A	0.9700
O1W—H1WB	0.8203	C18—H18B	0.9700
N1—C18	1.474 (3)	C20—H20A	0.9700
N1—C19	1.478 (3)	C20—H20B	0.9700
N1—H1A	0.9004	C8—H8A	0.9600
N1—H1B	0.9004	C8—H8B	0.9600
O1—C1	1.315 (3)	C8—H8C	0.9600
O1—H1	0.8206	C17—H17A	0.9700
O7—C20	1.411 (3)	C17—H17B	0.9700
O7—C17	1.412 (3)		
			
C11—C10—C15	117.5 (2)	C6—C5—C4	119.7 (2)
C11—C10—C9	121.9 (2)	C5—O3—C8	118.2 (2)
C15—C10—C9	120.5 (2)	C2—C7—C6	121.5 (2)
C13—O6—C16	118.19 (19)	C2—C7—H7A	119.3
C13—C12—C11	119.6 (2)	C6—C7—H7A	119.3
C13—C12—H12A	120.2	C3—C4—C5	120.8 (2)
C11—C12—H12A	120.2	C3—C4—H4A	119.6
C15—C14—C13	119.9 (2)	C5—C4—H4A	119.6
C15—C14—H14A	120.1	C5—C6—C7	119.3 (2)
C13—C14—H14A	120.1	C5—C6—H6A	120.3
O6—C13—C12	125.0 (2)	C7—C6—H6A	120.3
O6—C13—C14	115.1 (2)	C4—C3—C2	120.1 (2)
C12—C13—C14	119.8 (2)	C4—C3—H3A	120.0
C14—C15—C10	121.5 (2)	C2—C3—H3A	120.0
C14—C15—H15A	119.2	N1—C19—C20	109.8 (2)
C10—C15—H15A	119.2	N1—C19—H19A	109.7
C10—C11—C12	121.7 (2)	C20—C19—H19A	109.7
C10—C11—H11A	119.1	N1—C19—H19B	109.7
C12—C11—H11A	119.1	C20—C19—H19B	109.7
O5—C9—O4	123.9 (2)	H19A—C19—H19B	108.2
O5—C9—C10	117.0 (2)	N1—C18—C17	110.0 (2)
O4—C9—C10	119.1 (2)	N1—C18—H18A	109.7
O6—C16—H16A	109.5	C17—C18—H18A	109.7
O6—C16—H16B	109.5	N1—C18—H18B	109.7
H16A—C16—H16B	109.5	C17—C18—H18B	109.7
O6—C16—H16C	109.5	H18A—C18—H18B	108.2
H16A—C16—H16C	109.5	O7—C20—C19	112.0 (2)
H16B—C16—H16C	109.5	O7—C20—H20A	109.2
H1WA—O1W—H1WB	108.6	C19—C20—H20A	109.2
C18—N1—C19	110.12 (19)	O7—C20—H20B	109.2
C18—N1—H1A	109.9	C19—C20—H20B	109.2
C19—N1—H1A	109.6	H20A—C20—H20B	107.9
C18—N1—H1B	109.0	O3—C8—H8A	109.5
C19—N1—H1B	106.9	O3—C8—H8B	109.5
H1A—N1—H1B	111.3	H8A—C8—H8B	109.5
C1—O1—H1	109.2	O3—C8—H8C	109.5
C20—O7—C17	109.6 (2)	H8A—C8—H8C	109.5
O2—C1—O1	122.8 (2)	H8B—C8—H8C	109.5
O2—C1—C2	122.7 (3)	O7—C17—C18	111.7 (2)
O1—C1—C2	114.5 (2)	O7—C17—H17A	109.3
C7—C2—C3	118.6 (2)	C18—C17—H17A	109.3
C7—C2—C1	118.9 (2)	O7—C17—H17B	109.3
C3—C2—C1	122.4 (2)	C18—C17—H17B	109.3
O3—C5—C6	124.1 (2)	H17A—C17—H17B	107.9
O3—C5—C4	116.2 (2)		

### Hydrogen-bond geometry (Å, °)

**Table:**

*D*—H···*A*	*D*—H	H···*A*	*D*···*A*	*D*—H···*A*
N1—H1A···O5^i^	0.90	1.75	2.628 (3)	164
N1—H1B···O2	0.90	1.96	2.837 (3)	163
O1—H1···O1W^i^	0.82	1.76	2.579 (2)	173
O1W—H1WA···O4	0.82	1.91	2.714 (2)	167
O1W—H1WB···O4^ii^	0.82	1.91	2.712 (3)	164

Symmetry codes: (i) *x*, −*y*+1/2, *z*−1/2; (ii) *x*, −*y*+3/2, *z*+1/2.
